# Phenotype changes and impaired function of dendritic cell subsets in patients with sepsis: a prospective observational analysis

**DOI:** 10.1186/cc7969

**Published:** 2009-07-15

**Authors:** Holger Poehlmann, Joerg C Schefold, Heidrun Zuckermann-Becker, Hans-Dieter Volk, Christian Meisel

**Affiliations:** 1 Department of Medical Immunology, Charité Universitätsmedizin Berlin, Campus Mitte, Chariteplatz 1, Berlin 10117, Germany; 2 Department of Nephrology and Intensive Care Medicine, Charité Universitätsmedizin Berlin, Campus Vichow Clinic, Augustenburger Platz 1, Berlin 13353, Germany; 3 Department of General Surgery, Charité Universitätsmedizin Berlin, Campus Mitte, Chariteplatz 1, Berlin 10117, Germany; 4 Berlin-Brandenburg Center for Regenerative Therapies, Charité Universitätsmedizin Berlin, Campus Virchow Clinic, Augustenburger Platz 1, Berlin 13353, Germany

## Abstract

**Introduction:**

Patients with sepsis often demonstrate severely impaired immune responses. The hallmark of this state of immunoparalysis is monocytic deactivation characterized by decreased human leukocyte antigen (HLA)-DR expression and reduced production of proinflammatory cytokines. Recently, diminished numbers of dendritic cells (DCs) were reported in patients with sepsis. However, little is known about DC phenotype and function in human sepsis. We therefore compared phenotypic and functional changes in monocyte and DC subsets in patients with sepsis and immunoparalysis.

**Methods:**

In a prospective observational analysis, 16 consecutive patients with severe sepsis and septic shock (age 59.2 ± 9.7 years, 13 male, Sequential Organ Failure Assessment score 6.1 ± 2.7) and immunoparalysis (monocytic HLA-DR expression < 5,000 antibodies/cell) and 16 healthy volunteers were included. Peripheral blood DC counts, HLA-DR expression and *ex vivo *cytokine production were evaluated in comparison with monocyte subsets over time.

**Results:**

At baseline, a profound reduction in the numbers of myeloid DCs (MDCs), plasmacytoid DCs (PDCs), and CD14^dim^CD16^positive ^monocytes was observed in sepsis whereas CD14^bright^CD16^negative ^and CD14^bright^CD16^positive ^monocyte numbers were increased. HLA-DR expression was reduced on all monocyte and DC subsets. Production of proinflammatory cytokines and intracellular cytokine staining in response to lipopolysaccharide and lipoteichoic acid was impaired in monocyte subsets and MDCs, whereas IL-10 secretion was increased. IFNα response by stimulated PDCs was significantly decreased compared with controls. At day 28, HLA-DR expression and cytokine production of DC and monocyte subsets remained lower in septic patients compared with controls.

**Conclusions:**

In sepsis, long-lasting functional deactivation is common to all circulating monocyte and DC subsets. In addition to decreased peripheral blood DC counts, functional impairment of antigen-presenting cells may contribute to an impaired antimicrobial defense in sepsis.

## Introduction

Sepsis is a major medical challenge with a high annual incidence rate. Despite improvements in critical care, however, the outcome from sepsis has improved little and mortality rates remain high [1-3]. Earlier, the prevailing theory was that mortality from sepsis largely is a consequence of an overwhelming host inflammatory response [4-6]. Failure of clinical trials targeting inflammatory mediators to improve the outcome from sepsis and recent insights prompted reconsideration of this concept [4-8]. Today, it is recognized that the host's immune response during sepsis changes over time, resulting in both inflammation and profound immunosuppression in the later course of the disease. Many patients surviving the early phase of sepsis therefore often show signs of severe immunosuppression [4-6,9-16].

A number of immune dysfunctions have been reported in sepsis, including apoptosis of T lymphocytes and B lymphocytes, altered cellular cytokine production, increased levels of the anti-inflammatory IL-10, impaired phagocytosis, monocyte deactivation with diminished major histocompatibility class II molecule expression, and altered response to microbial products [17-22]. The term immunoparalysis was proposed to describe the host's general inability to mount effective immune responses. We and other workers have demonstrated an association between low levels of the major histocompatibility complex class II molecule human leukocyte antigen (HLA)-DR on monocytes and the impairment of cellular immunity in sepsis, including decreased production of proinflammatory cytokines, impaired antigen presentation, and reduced *ex vivo *lymphocyte response to recall antigens [9,20,23,24]. Importantly, prolonged downregulation of monocytic HLA-DR was associated with an adverse outcome from sepsis [20,24]. Consequently, a number of clinical pilot trials aiming to reverse immunoparalysis via immunomodulatory strategies were recently performed [9,25,26].

In contrast to the extensively studied major population of classical CD14^bright ^monocytes, little is known about phenotypic and functional changes of CD16^positive ^(Fcγ receptor III) monocyte subsets in sepsis. In healthy individuals about 10 to 15% of circulating monocytes are CD16^positive ^cells, which express higher levels of HLA-DR and proinflammatory cytokines than CD16^negative ^monocytes after stimulation with microbial products. This CD16^positive ^subset has therefore been referred to as proinflammatory monocytes [27-29]. Although expansion of CD16^positive ^monocytes was shown in sepsis [30], it is currently unclear whether this subset undergoes functional deactivation similar to classical CD14^bright^CD16^negative ^monocytes in sepsis.

Dendritic cells (DCs) are the most potent antigen-presenting cells (APCs) and play a key role in linking innate and adaptive host immune responses to microorganisms. Distinct subsets of circulating DCs can be identified in peripheral blood, including myeloid dendritic cells (MDCs) and plasmacytoid dendritic cells (PDCs) [31]. Although arising from common precursor cells in the bone marrow, MDCs and PDCs are phenotypically and functionally different [32]. For example, PDCs but not MDCs express the receptor for dsDNA (Toll-like receptor (TLR) 9), while TLR4, the receptor for bacterial lipopolysaccharide (LPS), is restricted to MDCs [31]. Activation of MDCs by LPS via TLR4 results in the secretion of TNFα, IL-1β and IL-6, while PDCs secrete enormous amounts of IFNα after stimulation with the TLR9 ligand CpG oligonucleotides (ODN), and may play an important role in antiviral immunity [31,33].

Upon encountering microbial products, DCs undergo phenotypic and functional maturation and migrate to secondary lymphatic organs, where they induce adaptive T-cell responses. Compromised DC function was associated with increased disease severity and adverse outcome in animal models of sepsis [34-36]. Increased apoptosis of DCs has been demonstrated in spleens from patients with sepsis, and an early decrease in circulating DCs was shown to correlate with increased disease severity and mortality [37,38]. Data on functional changes in DCs in sepsis patients, however, remain scarce.

The aim of the present study was to determine and compare phenotypic differences and functional changes in different monocyte and DC subsets over time in patients with sepsis and immunoparalysis.

## Materials and methods

### Study population and protocol

Sixteen consecutive patients (13 men, age 59 ± 9.7 years) with severe sepsis or septic shock and immunoparalysis hospitalized in the surgical intensive care unit of a tertiary care academic centre were included between January 2004 and January 2005. Sixteen healthy volunteers (13 men, age 46 ± 11.4 years) served as controls.

During the study interval, a total of 22 intensive care unit patients were screened for the presence of immunoparalysis, and all patients who fulfilled the inclusion criteria entered the analysis. The following inclusion criteria applied: age > 18 years, presence of severe sepsis or septic shock [39], and presence of immunoparalysis (monocytic HLA-DR expression < 5,000 antibodies/cell). Hepatitis B or hepatitis C patients, HIV patients and patients receiving immunosuppressive drugs (for example, steroids) were excluded.

Disease severity was assessed daily using the Simplified Acute Physiology Score 2 and the Sequential Organ Failure Assessment score. Clinical data, microbiological data and 28-day mortality were recorded. Blood samples were collected on the day after enrolment (baseline) and at study day 28. Informed consent was achieved from the patient or respective representatives. The study was performed in adherence with the Declaration of Helsinki and was approved by the local ethics committee on human research.

### Media and reagents

For *ex vivo *cell culture, RPMI 1640 medium (PAA Laboratories, Pasching, Germany) was used. The medium was tested for low TNF-inducing capacity (TNFα release < 10 pg/ml) in heparinized whole blood samples from healthy controls. Endotoxin (LPS) from *Escherichia coli *(L-4516) was purchased from Sigma (Steinheim, Germany) and lipoteichoic acid (LTA) from *Staphylococcus aureus *(DSM 20233) was a kind gift from Dr S. Morath (Konstanz, Germany). Commercially available ODN CpG 2336 (ODN class A), CpG 2243 (control class A), CpG 2395 (ODN class C) and CpG 2137 (control class C) were used (Coley Pharmaceutical, Kanata, Canada).

### Determination of cytokine secretion by monocytes and dendritic cells

Heparinized blood was diluted 1:5 in RPMI without supplements and was incubated (6 hours, 37°C, 5% CO_2_) with 100 ng/ml LPS or 10 μg/ml LTA for cytokine measurement in the supernatants. For stimulation with ODN class A and ODN class C, peripheral blood mononuclear cells were isolated from heparinized venous blood samples by density gradient centrifugation using Ficoll-Paque (Pharmacia, Freiburg, Germany). Peripheral blood mononuclear cells were cultured at a concentration of 2 × 10^6 ^cells/ml in supplemented RPMI 1640 medium and were stimulated with 1 μg/ml ODN class A, ODN class C, ODN control class A or ODN control class C. After incubation (24 hours, 37°C, 5% CO_2_) supernatants were separated from cells for cytokine measurement. Quantification of HLA-DR on circulating monocytes was performed using a standardized flow cytometric assay, as described elsewhere [40].

For enumeration of DC subsets, 150 μl whole blood was stained with FITC-conjugated antibodies against lineage markers (lin1) (mixture of anti-CD3/CD14/CD16/CD19/CD20/CD56), anti-CD123-PE, anti-HLA-DR-PerCP and anti-CD33-APC (BD Biosciences, Heidelberg, Germany). PDCs were gated as lin1 ^-^CD123^+^HLA-DR^+ ^events, and MDCs as lin1 ^-^CD33^+^HLA-DR^+ ^events. After treatment with FACS Lysing Solution (BD Biosciences), at least 150 to 300 events per DC population were analyzed on a FACSCalibur using CellQuest^Pro ^(BD Biosciences) software. HLA-DR expression on DCs was measured as the mean fluorescence intensity. Absolute APC population frequencies were calculated as white blood cell counts multiplied by the ratio of the APC population over all leukocytes.

### Intracellular cytokine staining by flow cytometry

For flow cytometric measurement of intracellular cytokines, heparinized blood samples were diluted 1:1 in RPMI without supplements and were stimulated with 100 ng/ml LPS and 10 μg/ml Brefeldin A (Sigma) for 6 hours (37°C, 5% CO_2_). After stimulation, cells were washed and stained with anti-CD14-FITC, anti-HLA-DR-PerCP and anti-CD33-APC (BD Biosciences). Leukocytes were fixed and permeabilized with FACS Lysing Solution and FACS Perm2 (BD Biosciences), and were stained with anti-TNFα-PE, anti-IL-1β-PE, anti-IL-6-PE, anti-IL-10-PE (BD Biosciences) or murine IgG_1_-PE (Immunotech, Marseille, France) as control.

### Detection of cytokines, procalcitonin and C-reactive protein

Cytokine production was assayed in culture supernatants and plasma by ELISA. Commercial kits were used to determine IFNα (PBL Biomedical Laboratories, Piscataway, NJ, USA), TNFα, IL-1β, IL-6 and IL-10 in supernatants (R&D Systems, Minneapolis, MN, USA). IL-10 plasma levels were measured by ultrasensitive ELISA (lower detection limit, 0.78 pg/ml; Biosource, Nivelles, Belgium). Immunoluminometric assays (Lumi^® ^PCT; Brahms, Hennigsdorf, Germany) were used to detect procalcitonin plasma levels. High-sensitivity C-reactive protein was measured immunoturbidometrically in a certified laboratory.

### Statistical analysis

For statistical analyses, SPSS for Windows software (version 12.0; SPSS, Inc., Chicago, IL, USA) was used. Data are presented as the mean ± standard deviation. The Mann – Whitney U test was used for comparison between patients and controls. Wilcoxon's test was used for comparison between baseline and day 28 in the patient group. *P *< 0.05 was considered significant.

## Results

### Study population and follow-up

Sixteen consecutive patients with severe sepsis or septic shock and immunoparalysis were included (Table 1). The mean stay on the intensive care unit until inclusion was 3.2 ± 2.9 days and sepsis was diagnosed on the day of intensive care unit admission in all individuals. At inclusion, 10 patients had septic shock and received vasopressor (norepinephrine) support.

**Table 1:** Demographics of the study patients

Patient	Sex	Age (years)	28-day mortality	Site of infection	SAPS 2/SOFA score (baseline)	Disease severity	Cultures positive^a^
1	Male	62	Survivor	Pneumonia	45/5	Septic shock	*Enterococcus aerogenes*
2	Male	43	Nonsurvivor	Peritonitis	44/11	Septic shock	None
3	Male	64	Survivor	Necrotizing pancreatitis	38/7	Septic shock	*Enterococcus cloacae*
4	Male	54	Survivor	Peritonitis	52/2	Severe sepsis	Coagulase-negative Staphylococcus, *Candida albicans*
5	Male	60	Survivor	Mediastinitis	22/5	Severe sepsis	*Pseudomonas aeruginosa*, *Enterococcus faecium*
6	Male	75	Survivor	Pneumonia	42/8	Septic shock	*Escherichia coli*
7	Female	59	Survivor	Peritonitis	33/4	Severe sepsis	*Enterococcus faecalis*
8	Male	64	Nonsurvivor	Pneumonia	30/4	Septic shock	*E. cloacae*
9	Male	46	Survivor	Necrotizing pancreatitis	29/11	Septic shock	*P. aeruginosa*, *E. faecalis*, coagulase-negative Staphylococcus
10	Male	66	Survivor	Pneumonia	29/7	Septic shock	*Klebsiella oxytoca*
11	Female	53	Survivor	Pneumonia	33/9	Septic shock	*Klebsiella pneumoniae*
12	Male	52	Survivor	Peritonitis	24/3	Septic shock	*E. coli*, *P. aeruginosa*
13	Male	54	Survivor	Mediastinitis	23/8	Severe sepsis	*E. faecalis*
14	Male	65	Nonsurvivor	Pneumonia	30/5	Severe sepsis	*E. aerogenes*
15	Male	79	Survivor	Graft infection	33/4	Severe sepsis	Coagulase-negative Staphylococcus., *E. faecium*
16	Female	51	Survivor	Necrotizing pancreatitis	29 5	Septic shock	*C. albicans*, *Staphylococcus aureus*

SAPS 2, Simplified Acute Physiology Score 2; SOFA, Sequential Organ Failure Assessment. ^a^Isolated microbes at the respective site of infection.

Disease severity scores improved slightly from baseline to day 28 (Simplified Acute Physiology Score 2, 33.5 ± 8.5 to 28.8 ± 11.9, not significant; Sequential Organ Failure Assessment score, 6.1 ± 2.7 to 4.8 ± 2.2, *P *< 0.05). The mean procalcitonin and C-reactive protein levels decreased from baseline to day 28 (procalcitonin, 2,924 ± 3,860 to 445 ± 470 pg/ml, *P *< 0.01; C-reactive protein, 20.2 ± 9.4 to 10.6 ± 5.8 mg/dl, *P *< 0.01). IL-10 levels also decreased over time (20.2 ± 53.4 to 4.0 ± 2.0 pg/ml, *P *< 0.05).

The major etiology of sepsis was pneumonia (n = 6), peritonitis (n = 4), and pancreatitis (n = 3) (Table 1). In 15/16 patients, positive cultures (n = 8 Gram-negative, n = 4 mixed spectrum) of relevant microorganisms were recorded (Table 1). Gram-negative infection was associated with the presence of septic shock (*P *< 0.05). The 28-day mortality rate was 19%.

### Distribution of monocyte and dendritic cell subsets in sepsis

**Figure 1:**
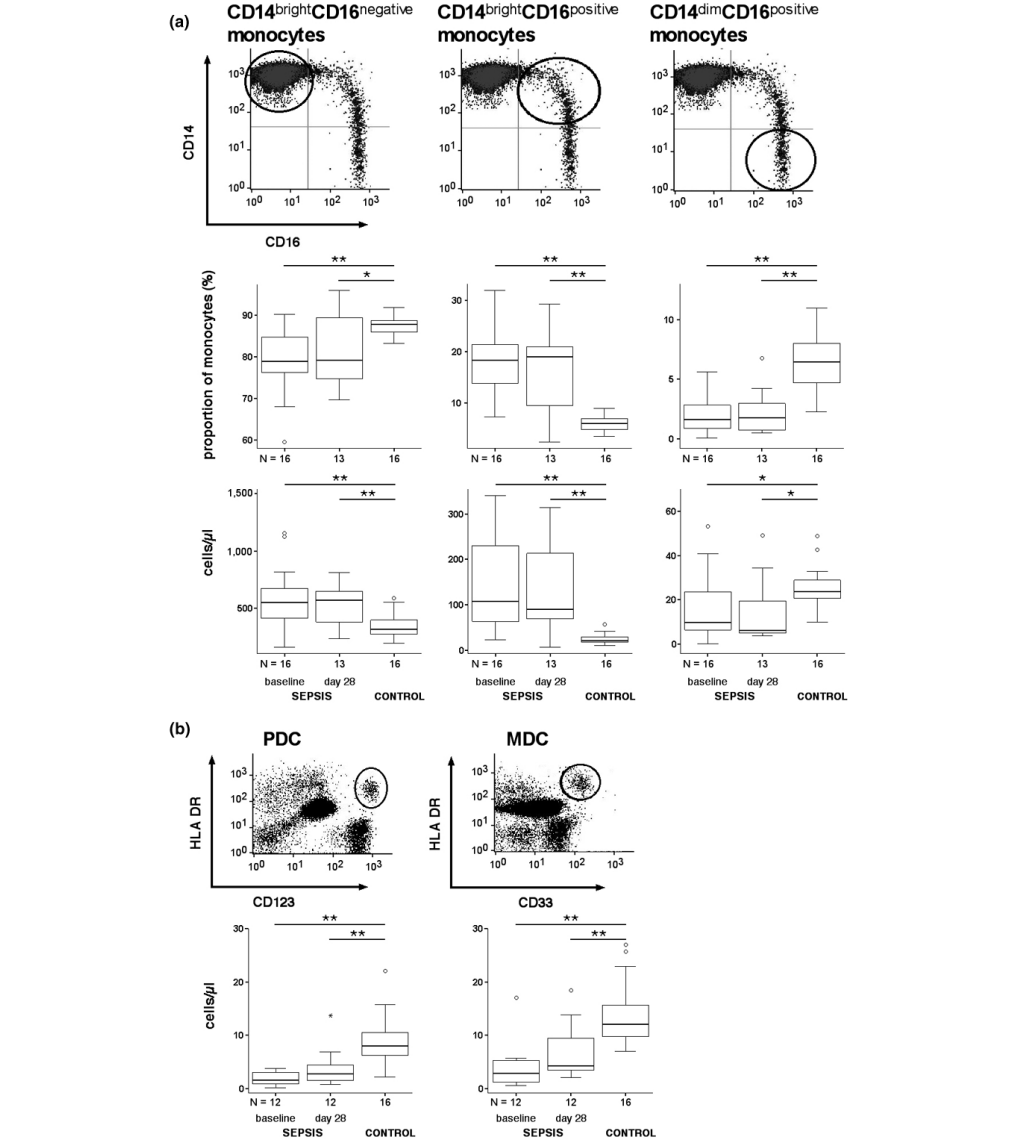
**Circulating monocyte and dendritic cell subset frequencies in sepsis patients**. Frequencies of circulating monocyte and dendritic cell subsets in patients with sepsis at baseline and at day 28 compared with controls. **(a) **Monocytes were gated using a forward scatter/side scatter plot and a CD14/CD33 scatter plot, where CD33^negative ^cells (lymphocytes and plasmacytoid dendritic cells (PDCs)) as well as CD14^negative^CD33^bright ^myeloid dendritic cells (MDCs) were excluded from further analysis. According to their differential expression of CD14 and CD16, three monocyte subsets were defined: CD14^bright^CD16^negative^, CD14^bright^CD16^positive ^and CD14^dim^CD16^positive^. Proportions and cell counts of each subset are given. **(b) **Dendritic cell subsets were defined as follows: PDCs were gated as lineage marker (CD3, CD14, CD16, CD19, CD20, CD56)-negative (lin^-^) CD123^+^human leukocyte antigen (HLA)-DR^+ ^cells, and MDCs as lin ^-^CD33^+^HLA-DR^+ ^cells. Absolute cell counts are given. *P < 0.05, **P < 0.01.

Three different monocyte subsets can be distinguished in peripheral blood according to their CD14 and CD16 surface expression [41] (Figure 1a and Table 2). At baseline, the frequency of CD14^bright^CD16^positive ^monocytes was increased in sepsis patients compared with healthy controls (17.9 ± 6.2% vs. 6.0 ± 1.6%, *P *< 0.001; Figure 1a). In contrast, the proportion of CD14^dim^CD16^positive ^monocytes decreased in sepsis patients (3.0 ± 5% vs. 6.4 ± 2.5% (controls), *P *< 0.001). The opposing changes in the two CD16^positive ^monocyte subsets were also observed for absolute cell numbers, along with the typical increase in CD14^bright^CD16^negative ^monocytes (Figure 1a). At day 28, cell counts of all monocyte subsets remained significantly different from controls (Figure 1a).

**Table 2:** Monocyte and dendritic cell subsets

Monocyte or dendritic cell subset	Surface marker expression	Properties
Classical monocytes	CD14^bright^CD16^negative^	Majority of circulating monocytes [27-29]^9^
Inflammatory monocytes	CD14^bright^CD16^positive^	Produce high level of proinflammatory cytokines, increased in sepsis [27-29]
Dendritic cell-like monocytes	CD14^dim^CD16^positive^	Morphological and functional similarities to dendritic cells [42,43]
Plasmacytoid dendritic cells	lin1 ^-^CD123^+^HLA-DR^+^	Produce high level of IFNα in response to viruses [31-35]
Myeloid dendritic cells	lin1 ^-^CD33^+^HLA-DR^+^	Potent antigen-presenting cells [31-35]

lin1, lineage marker.

Human peripheral blood contains at least two distinct populations of DCs [42]. In the present study, PDCs (lin1^-^CD123^+^HLA-DR^+^) and MDCs (lin1 ^-^CD33^+^HLA-DR^+^) were analyzed (Figure 1b). Compared with controls, both PDC and MDC counts in sepsis patients were lower at baseline (PDCs, 8.6 ± 4.0 vs. 1.9 ± 1.3 cells/μl, *P *< 0.001; MDCs, 13.5 ± 5.5 vs. 4.2 ± 4.5 cells/μl, *P *< 0.001; Figure 1b). Both DC populations recovered slightly until day 28 (PDCs, 3.8 ± 3.6 cells/μl; MDCs, 6.7 ± 5.1 cells/μl), but remained below corresponding control levels (both *P *< 0.001; Figure 1b).

### HLA-DR expression on monocyte and dendritic cell subsets

**Figure 2:**
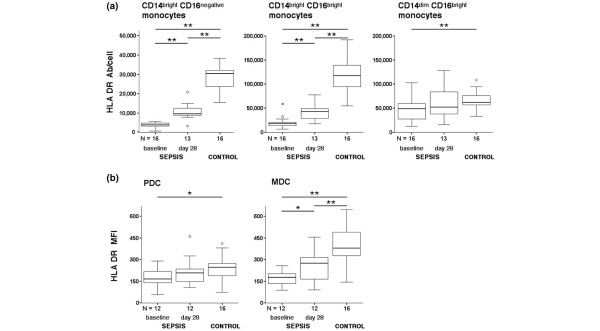
**Diminished human leukocyte antigen-DR expression on circulating monocyte and dendritic cell subsets in sepsis patients**. Diminished human leukocyte antigen (HLA)-DR expression on circulating monocyte and dendritic cell subsets in patients with sepsis at baseline and at day 28 compared with healthy controls. Monocyte and dendritic cell subsets were stained and gated as described in Figure 1. **(a) **The HLA-DR expression in each monocyte subset was quantified using a standardized assay as described in Materials and methods, and is given as HLA-DR antibodies per cell (Ab/cell). **(b) **For plasmacytoid dendritic cells (PDCs) and myeloid dendritic cells (MDCs), the HLA-DR expression is given as the mean fluorescence intensity (MFI). *P < 0.05, **P < 0.01.

Reduced monocytic HLA-DR expression is a hallmark of immunoparalysis. While previous studies have focused on HLA-DR expression on classical monocytes (CD14^bright^CD16^negative^), little is known about the regulation of HLA-DR on CD16^positive ^monocyte and DC subsets in sepsis. In line with the inclusion criteria, HLA-DR on CD14^bright^CD16^negative ^monocytes was strongly diminished in patients compared with controls (*P *< 0.001; Figure 2a). At day 28, HLA-DR expression on CD14^bright^CD16^negative ^monocytes increased (*P *< 0.01) but remained below control levels (*P *< 0.001; Figure 2a). Only three patients reached persistent monocytic HLA-DR levels above the lower normal range (> 15,000 antibodies/cell), indicating recovery from immunoparalysis. No significant association between severity of immunoparalysis (that is, monocytic HLA-DR levels) and clinical outcome of patients was observed.

Similar to classical monocytes, HLA-DR expression on the CD14^bright^CD16^positive ^monocyte subset was approximately eightfold reduced in comparison with controls (*P *< 0.001) at baseline, and remained significantly different from control levels at day 28 (Figure 2a). In contrast, HLA-DR downregulation on CD14^dim^CD16^positive ^monocytes was less pronounced (1.5-fold compared with controls at baseline, *P *< 0.01) and HLA-DR levels did not statistically differ from controls at day 28 (Figure 2a).

We found a significant downregulation of HLA-DR on both DC subsets in septic patients that was more pronounced on MDCs (Figure 2b): HLA-DR on MDCs averaged 174 ± 54 mean fluorescence intensity in sepsis at baseline and 497 ± 128 mean fluorescence intensity in controls (*P *< 0.001). This almost threefold reduction was not seen on PDCs, which showed an expression of 177 ± 66 mean fluorescence intensity at baseline compared with 239 ± 77 mean fluorescence intensity in controls (*P *< 0.05). At day 28, HLA-DR expression on MDCs slightly recovered (257 ± 105 mean fluorescence intensity) but remained significantly reduced when compared with controls (*P *< 0.01), while HLA-DR levels on PDCs (216 ± 97 mean fluorescence intensity) almost reached control levels (Figure 2b).

### Cytokine secretion by monocytes and dendritic cells

**Figure 3:**
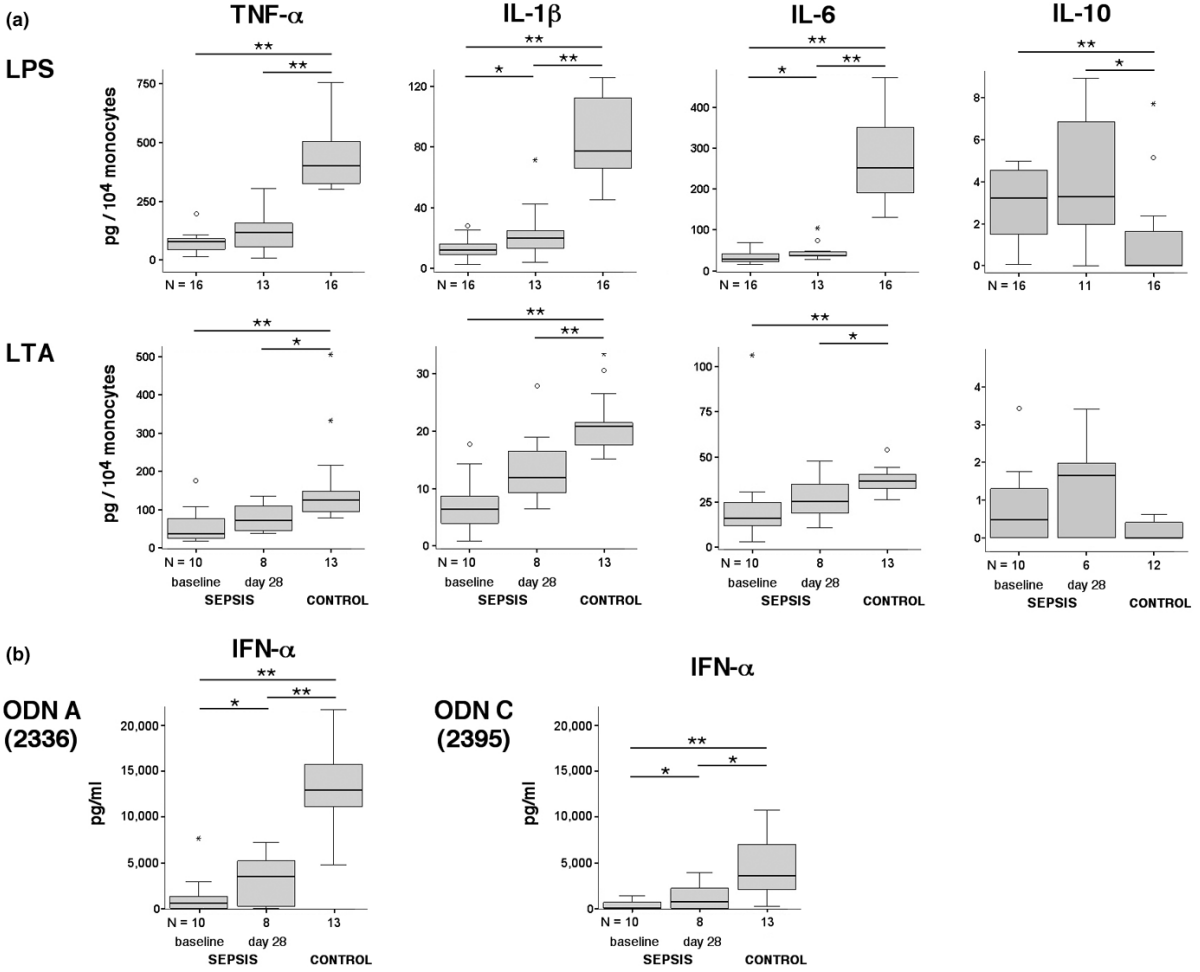
**Cytokine production after stimulation of blood from sepsis patients using different Toll-like receptor ligands**. Cytokine production after stimulation of whole blood and peripheral blood mononuclear cells from patients with sepsis using different Toll-like receptor ligands. **(a) **Blood was drawn from healthy controls or patients at the indicated time points (baseline, day 28) and stimulated for 6 hours with lipopolysaccharide (LPS) or lipoteichoic acid (LTA). Cytokine levels were measured in the supernatants by ELISA and were normalized to monocyte counts. **(b) **Simultaneously, peripheral blood mononuclear cells were stimulated with either class A (2336) or class C (2395) oligonucleotides (ODN). After 24 hours, the concentration of IFNα in the supernatants was determined using ELISA. *P < 0.05, **P < 0.01.

To determine the functional status of APC subsets, we assessed the cellular cytokine secretion profile. First, the production of TNFα, IL-1β, IL-6 and IL-10 was analyzed in whole blood and peripheral blood mononuclear cell cultures after stimulation with TLR ligands. Secretion of proinflammatory cytokines in cultures from septic patients was significantly diminished at both time points compared with cultures from controls (Figure 3a). In contrast, IL-10 secretion after LPS stimulation was significantly enhanced at baseline and day 28. Stimulation with LTA exhibited less biological potency than LPS, but revealed similar findings with significantly reduced levels of all three proinflammatory cytokines and a tendency towards increased IL-10 secretion in whole blood cultures from septic patients.

The functionality of PDCs was assessed via TLR9 stimulation using CpG ODN. Three different classes of ODN (class A, class B, class C) have been identified and linked to preferential cytokine induction in either PDCs (class A) or B cells (class B), or both (class C). Because activation of TLR9 signaling by ODN in PDCs, but not in B cells, has been shown to specifically induce secretion of IFNα, we used class A and class C ODN for stimulation of peripheral blood mononuclear cells to determine IFNα secretion by PDCs. At baseline, IFNα secretion was depressed after stimulation with ODN class A (*P *< 0.001) as well as ODN class C (*P *< 0.001) when compared with controls. A significant recovery was observed at day 28 versus baseline for both ODN class A stimulation (*P *< 0.05) and ODN class C stimulation (*P *< 0.05), but IFNα levels remained lower than in controls (*P *< 0.001 and *P *< 0.05 for ODN class A and ODN class C, respectively). Reduced IFNα secretion is unlikely to be only due to the observed decrease in PDC counts, since IFNα release at baseline is decreased about ninefold and 11-fold for ODN class A and ODN class C, respectively, compared with controls whereas PDC counts were diminished by only fourfold. Similarly, PDC counts at day 28 in septic patients were twofold lower compared with controls, but IFNα secretion was reduced by fourfold for both ODN class A and ODN class C (Figures 2b and 3b).

### Intracellular levels of cytokines in monocyte subsets and myeloid dendritic cells

To characterize the diminished cytokine production at the cellular level, APC subsets were analyzed by intracellular cytokine staining. Since identification of CD16^positive ^monocyte subsets is impaired by the loss of CD16 expression shortly after LPS stimulation (data not shown), CD33 was used as an alternative marker. Classical CD14^bright^CD16^negative ^and CD14^bright^CD16^positive ^monocytes express high levels of CD33, whereas CD14^dim^CD16^positive ^monocytes are CD33^dim^. This remains unchanged during LPS stimulation (data not shown) and enabled us to evaluate classical CD14^bright^CD33^bright ^monocytes versus CD14^dim^CD33^dim^(CD16^bright^) monocytes. MDCs were identified by the lack of CD14 and higher CD33 expression compared with CD14^bright ^monocytes.

**Figure 4:**
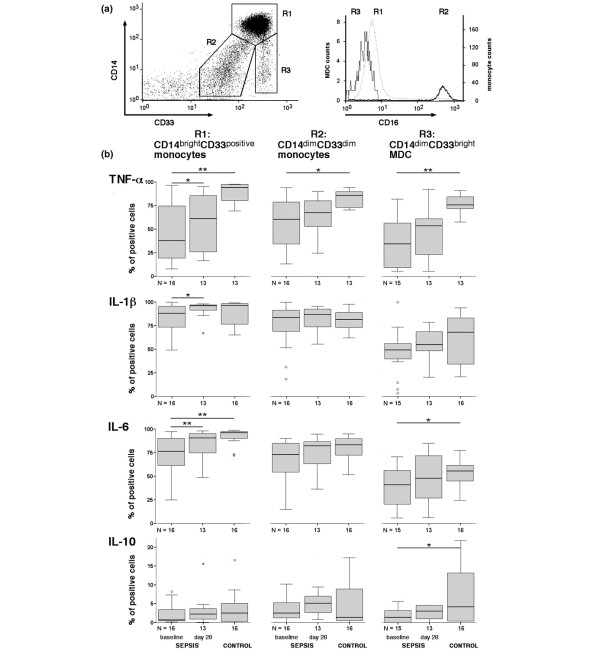
**Lipopolysaccharide-induced intracellular cytokine expression in monocytes and myeloid dendritic cells in sepsis patients**. Lipopolysaccharide (LPS)-induced intracellular cytokine expression in monocytes and in myeloid dendritic cells (MDCs) in patients with sepsis compared with healthy controls. **(a) **Unstimulated blood was stained for CD14, CD33 and CD16, and then monocyte subsets (region (R) 1, CD14^bright^CD33^bright^; R2, CD14^dim^CD33^dim^) and MDCs (R3, CD14^dim^CD33^bright^) were gated in a CD14/CD33 scatter plot for subsequent analysis of CD16 expression. This staining strategy allowed identification of CD16^positive ^(CD14^dim^CD33^dim^) monocytes (R2) despite the loss of CD16 expression after LPS stimulation. **(b) **Blood was drawn from healthy controls and from sepsis patients, and was stimulated with LPS (6 hours) in the presence of the secretion blocker Brefeldin A. After surface staining for CD14, HLA-DR and CD33, cells were intracellularly stained for TNFα, IL-1β, IL-6, and IL-10 using PE-labeled cytokine-specific monoclonal antibody or murine IgG_1_-PE as control. The percentage of cytokine-positive CD14^bright^CD33^positive ^monocytes, CD14^dim^CD33^dim ^monocytes and CD14^negative^CD33^bright ^MDCs is given. *P < 0.05, **P < 0.01.

At baseline, the percentage of TNFα-producing (*P *< 0.01) and IL-6-producing (*P *< 0.01) CD14^bright ^monocytes was diminished after LPS stimulation in septic patients compared with controls, whereas no significant differences were observed for IL-1β and IL-10 producers (Figure 4). An increased proportion of TNFα-positive cells (*P *< 0.05), IL-1β-positive cells (*P *< 0.05) and IL-6-positive cells (*P *= 0.01) was observed at day 28 compared with baseline (Figure 4). In the CD33^dim^(CD16^positive^) monocyte population, a similar reduction in the percentage of cytokine-producing cells was observed at baseline, although this reached statistical significance only for TNFα (*P *< 0.05 vs. controls; Figure 4). In MDCs, the frequency of TNFα-producing cells (*P *< 0.005), IL-6-producing cells (*P *< 0.05) and IL-10-producing cells (*P *< 0.05) was significantly impaired at baseline compared with controls (Figure 4).

At day 28, the percentage of cytokine-positive cells after LPS stimulation was not significantly different (compared with controls) for all subsets despite strongly reduced cytokine levels in supernatants of LPS-stimulated or LTA-stimulated whole blood cultures (Figures 3 and 4).

## Discussion

Altered monocyte function, including diminished HLA-DR expression and impaired proinflammatory cytokine response, was previously reported in patients with sepsis, severe trauma and major surgery. Such monocytic deactivation indicates a state of globally impaired immune functions and correlates with poor clinical outcome in critically ill patients. Nevertheless, whether this phenomenon is restricted to classical monocytes or is common to all monocyte and DC subsets is currently unclear. We demonstrate that sepsis-induced immune dysfunction affects all circulating myeloid APC subsets and that these functional alterations are long-lasting.

Today, it is well established that circulating monocytes represent a heterogeneous cell population. Among the antigenic markers, CD14 and CD16 (also known as Fcγ RIII) are commonly used to distinguish monocyte subsets (Table 2). In addition to the majority of monocytes that express high levels of CD14 but not CD16, a minor population of CD16^positive ^monocytes was identified. These cells have characteristic expression patterns distinct from classical monocytes, including high HLA-DR expression. CD16^positive ^monocytes may be subdivided into CD14^bright ^and CD14^dim ^cells. The latter subset has morphological and functional similarities to DCs, including a strong capacity to activate naïve T cells *in vitro *[42,43]. Expansion of CD14^bright^CD16^positive ^monocytes has been observed in patients with sepsis and other inflammatory conditions [30,42,43]. Little is known, however, of the functional changes including both cytokine production and HLA-DR expression in CD16^negative ^and CD16^positive ^monocyte subsets during the course of sepsis.

Consistent with previous reports, we observed a significant increase in the circulating numbers of CD14^bright^CD16^negative ^and CD14^bright^CD16^positive ^monocytes in sepsis. Unlike these monocyte subsets, CD14^dim^CD16^positive ^monocytes were significantly decreased in our patient population. This is in contrast with previous data demonstrating an increase in both CD14^bright ^and CD14^dim^CD16^positive ^monocytes in neonates and children with sepsis [44], and may reflect age-related differences in the differentiation and/or survival of CD16^positive ^monocytes.

Similar to classical monocytes, we observed that CD16^positive ^subsets show signs of profound functional deactivation in sepsis. Although HLA-DR levels differ between respective subsets, HLA-DR was diminished in all monocyte subsets in sepsis at baseline. Notably, CD14^dim^CD16^positive ^monocytes showed only a slight reduction of HLA-DR expression at baseline while HLA-DR levels of both CD14^bright ^subsets remained significantly diminished in sepsis even at day 28. Moreover, consistent with previous data [9,45], we observed significantly reduced proinflammatory cytokine production (TNFα, IL-1β, IL-6) and increased anti-inflammatory cytokine levels (IL-10) after stimulation of whole blood with LPS and LTA in septic patients. Although we did not determine cytokine secretion in isolated monocyte subsets, we demonstrate reduced intracellular levels of TNFα and IL-6 in both CD16^negative ^and CD16^positive ^monocytes after LPS stimulation. Together with markedly diminished cytokine levels in the supernatants of LPS-stimulated whole blood cultures from septic patients (despite a significant increase in absolute numbers), this may indicate that both CD16^negative ^and CD16^positive ^monocytes undergo deactivation in sepsis.

Interestingly, in contrast to the markedly reduced cytokine levels in supernatants of stimulated whole blood cultures, differences in the percentage of cytokine-positive monocytes after LPS stimulation were less pronounced between patients and controls. Notably, the percentage of IL-1β-positive monocytes did not differ between septic patients and controls even at baseline, suggesting that proteolytic processing and/or secretion of IL-1β rather than synthesis and intracellular accumulation of inactive pro-IL-1β in monocytes are defective in sepsis. In fact, interference with the proteolytic cleavage of pro-IL-1β and secretion of mature IL-1β was proposed as a potential mechanism of the immunosuppressive effect of IL-10 [46]. In addition to defects in cytokine transcription and translation, reduced monocytic cytokine production in sepsis may result from impaired post-translational processes involved in cytokine secretion.

DCs are key players in innate and adaptive immune responses. During infection, tissue-resident DCs recognize characteristic microbial patterns resulting in the uptake of pathogens, maturation and migration of DCs to lymphoid tissue, and activation of T-cell responses. In mice, previous studies demonstrated extensive depletion of DCs in secondary lymphatic organs after endotoxin challenge and polymicrobial sepsis [34,47,48]. Markedly reduced numbers of DCs were also observed in the spleens of patients with sepsis [38]. In a mouse model of polymicrobial sepsis induced by cecal ligation and puncture, increased numbers of apoptotic CD11c^+ ^DCs in mesenteric lymph nodes have been demonstrated as early as 24 hours after cecal ligation and puncture [34]. Moreover, reduced numbers of circulating DCs in patients have been observed within 24 hours after onset of septic shock [37]. These data indicate that DC apoptosis occurs early in sepsis, and prompted us to assess functional DC alterations in the course of sepsis. We observed a profound reduction in peripheral MDC and PDC counts in septic patients at baseline that remained significantly decreased on day 28 compared with controls. Whether this is due to ongoing DC apoptosis, due to increased migration of circulating precursor DCs into peripheral sites of inflammation or results from prolonged diminished re-population of DCs from the bone marrow, however, remains speculative [49].

To the best of our knowledge, we are the first to demonstrate a marked and sustained functional impairment of circulating DC subsets in patients with sepsis. Similar to the phenotypic changes in monocytes resembling functional deactivation, peripheral blood DCs from septic patients showed a downregulation of surface HLA-DR expression and a reduced secretion of proinflammatory cytokines upon stimulation with microbial products. Relative to healthy controls, stimulation of MDCs from septic patients with LPS resulted in significantly reduced production of TNFα and IL-6, as indicated by intracellular cytokine staining. In addition, IFNα secretion by PDCs after stimulation with TLR9-activating CpG ODN was significantly decreased in sepsis, and this reduction exceeded (more than twofold) the decrease in PDC counts both at baseline and after 28 days.

Collectively, our data suggest a functional deactivation of both MDCs and PDCs during sepsis. Recent experimental findings provided experimental evidence for a crucial role of defective DC responses for the increased susceptibility to secondary infections during sepsis by demonstrating that increased mortality to an otherwise innocuous pulmonary *Aspergillus fumigatus *or *Pseudomonas aeruginosa *challenge in post-septic mice can be reversed by adoptive therapy using bone-marrow-derived DCs [49,50]. Moreover, significantly lower peripheral blood MDC and PDC counts have already been observed in nonsurvivors early after onset of septic shock [37]. Whether the loss of DC correlated to the persistence of primary infections or to the occurrence of opportunistic infections, however, was not investigated. Further studies are needed to elucidate the specific consequences of the sustained loss and dysfunction of circulating DC subsets for impaired antimicrobial defenses in sepsis patients.

The mechanisms leading to altered cytokine responses and diminished major histocompatibility complex class II expression in DCs during sepsis are incompletely understood. Recent experimental studies *in vitro *and *in vivo *have demonstrated that DCs, similar to monocytes, become tolerant after exposure to microbial products – resulting in reduced production of proinflammatory cytokines upon repeated stimulation [48,51]. In addition to impaired cytokine responses, endotoxin-desensitized DCs were shown to be poor inducers of T-helper type 1 cell responses [51]. Tolerance induction in DCs was also shown for other TLR ligands, including CpG ODN [52]. The data presented here are consistent with a functional impairment of TLR4 and TLR9 agonist-induced cellular responses in MDCs and PDCs in patients with sepsis. In line with previous findings, we found increased IL-10 levels in septic patients at baseline and at day 28 [17,21]. IL-10 might contribute to the observed downregulation of HLA-DR on monocytes and DCs via enhanced re-endocytosis and sequestration of HLA-DR in monocytes [17]. In line with recent findings demonstrating that IL-10 inhibits IFNα production in PDCs *in vitro*, we observed in our study an inverse correlation of IL-10 levels with CpG-induced IFNα production by PDCs (*P *< 0.001, Spearman rho = -0.83). Nonetheless, other factors including downregulation of TLR-receptor expression and altered TLR-induced signal transduction may play a role in monocyte and DC deactivation in sepsis [48].

## Conclusions

We demonstrate a profound and sustained functional impairment of all circulating monocyte and DC subsets in patients with sepsis and immunoparalysis. Further studies are required to elucidate the individual role of different APC populations in the compromised antimicrobial defenses in sepsis, and to determine whether reconstitution of APC function by immunomodulatory interventions may improve sepsis outcome.

## Key messages

• Patients with severe sepsis or septic shock show profound deactivation of *all *circulating monocyte and DC subsets, including CD16^positive ^monocytes, in MDCs and PDCs.

• Phenotypic and functional changes of monocyte and DC subsets include reduced major histocompatibility complex class II expression and diminished production of proinflammatory cytokines, whereas IL-10 secretion is increased.

• Functional changes in monocytes and DCs are long-lasting.

• Further studies are required to elucidate the individual role of different APC populations for compromised antimicrobial defenses in sepsis.

## Abbreviations

APC: antigen-presenting cell; DC: dendritic cell; ELISA: enzyme-linked immunosorbent assay; FITC: Fluoresceinisothiocyanat; HLA: human leukocyte antigen; IFN: interferon; IL: interleukin; LPS: lipopolysaccharide or endotoxin; LTA: lipoteichoic acid; MDC: myeloid dendritic cell; ODN: oligonucleotides; PDC: plasmacytoid dendritic cell; PE: phycoerythrin; TLR: Toll-like receptor; TNF: tumor necrosis factor.

## Competing interests

The authors declare that they have no competing interests.

## Authors' contributions

H-DV and CM were responsible for the study design. HP performed all experiments and recorded all clinical data. HP, JCS, and CM were responsible for data management and statistical analysis. HP, JCS and CM wrote the manuscript. HZ-B was responsible for patient recruitment and management, and participated together with H-DV in the interpretation of all data and revised the manuscript for important intellectual content.

## References

[B1] Angus DC Pereira CA Silva E Epidemiology of severe sepsis around the world Endocr Metab Immune Disord Drug Targets 2006 6 207 212 16787296 10.2174/187153006777442332

[B2] Brun-Buisson C Meshaka P Pinton P Vallet B EPISEPSIS: a reappraisal of the epidemiology and outcome of severe sepsis in French intensive care units Intensive Care Med 2004 30 580 588 14997295 10.1007/s00134-003-2121-4

[B3] Engel C Brunkhorst FM Bone HG Brunkhorst R Gerlach H Grond S Gruendling M Huhle G Jaschinski U John S Mayer K Oppert M Olthoff D Quintel M Ragaller M Rossaint R Stuber F Weiler N Welte T Bogatsch H Hartog C Loeffler M Reinhart K Epidemiology of sepsis in Germany: results from a national prospective multicenter study Intensive Care Med 2007 33 606 618 17323051 10.1007/s00134-006-0517-7

[B4] Annane D Bellissant E Cavaillon JM Septic shock Lancet 2005 365 63 78 15639681 10.1016/S0140-6736(04)17667-8

[B5] Cohen J The immunopathogenesis of sepsis Nature 2002 420 885 891 12490963 10.1038/nature01326

[B6] Hotchkiss RS Karl IE The pathophysiology and treatment of sepsis N Engl J Med 2003 348 138 150 12519925 10.1056/NEJMra021333

[B7] Carlet J Cohen J Calandra T Opal SM Masur H Sepsis: time to reconsider the concept Crit Care Med 2008 36 964 966 18431286 10.1097/CCM.0B013E318165B886

[B8] Schefold JC Hasper D Reinke P Monneret G Volk HD Consider delayed immunosuppression into the concept of sepsis [Letter] Crit Care Med 2008 36 3118 18941324 10.1097/CCM.0b013e31818bdd8f

[B9] Docke WD Randow F Syrbe U Krausch D Asadullah K Reinke P Volk HD Kox W Monocyte deactivation in septic patients: restoration by IFN-gamma treatment Nat Med 1997 3 678 681 9176497 10.1038/nm0697-678

[B10] Ertel W Kremer JP Kenney J Steckholzer U Jarrar D Trentz O Schildberg FW Downregulation of proinflammatory cytokine release in whole blood from septic patients Blood 1995 85 1341 1347 7858264

[B11] Fumeaux T Dufour J Stern S Pugin J Immune monitoring of patients with septic shock by measurement of intraleukocyte cytokines Intensive Care Med 2004 30 2028 2037 15368036 10.1007/s00134-004-2429-8

[B12] Munford RS Pugin J Normal responses to injury prevent systemic inflammation and can be immunosuppressive Am J Respir Crit Care Med 2001 163 316 321 11179099 10.1164/ajrccm.163.2.2007102

[B13] Oberholzer A Oberholzer C Moldawer LL Sepsis syndromes: understanding the role of innate and acquired immunity Shock 2001 16 83 96 11508871 10.1097/00024382-200116020-00001

[B14] Schefold JC Hasper D Volk HD Reinke P Sepsis: time has come to focus on the later stages Med Hypotheses 2008 71 203 208 18448264 10.1016/j.mehy.2008.03.022

[B15] Volk HD Reinke P Docke WD Clinical aspects: from systemic inflammation to 'immunoparalysis' Chem Immunol 2000 74 162 177 10608087 10.1159/000058753

[B16] Volk HD Reinke P Krausch D Zuckermann H Asadullah K Muller JM Docke WD Kox WJ Monocyte deactivation – rationale for a new therapeutic strategy in sepsis Intensive Care Med 1996 22 Suppl 4 S474 S481 8923092 10.1007/BF01743727

[B17] Fumeaux T Pugin J Role of interleukin-10 in the intracellular sequestration of human leukocyte antigen-DR in monocytes during septic shock Am J Respir Crit Care Med 2002 166 1475 1482 12406851 10.1164/rccm.200203-217OC

[B18] Hotchkiss RS Tinsley KW Swanson PE Schmieg RE Jr Hui JJ Chang KC Osborne DF Freeman BD Cobb JP Buchman TG Karl IE Sepsis-induced apoptosis causes progressive profound depletion of B and CD4^+ ^T lymphocytes in humans J Immunol 2001 166 6952 6963 11359857 10.4049/jimmunol.166.11.6952

[B19] Monneret G Finck ME Venet F Debard AL Bohe J Bienvenu J Lepape A The anti-inflammatory response dominates after septic shock: association of low monocyte HLA-DR expression and high interleukin-10 concentration Immunol Lett 2004 95 193 198 15388260 10.1016/j.imlet.2004.07.009

[B20] Monneret G Lepape A Voirin N Bohe J Venet F Debard AL Thizy H Bienvenu J Gueyffier F Vanhems P Persisting low monocyte human leukocyte antigen-DR expression predicts mortality in septic shock Intensive Care Med 2006 32 1175 1183 16741700 10.1007/s00134-006-0204-8

[B21] Randow F Syrbe U Meisel C Krausch D Zuckermann H Platzer C Volk HD Mechanism of endotoxin desensitization: involvement of interleukin 10 and transforming growth factor beta J Exp Med 1995 181 1887 1892 7722463 10.1084/jem.181.5.1887 PMC2191997

[B22] Wesche DE Lomas-Neira JL Perl M Chung CS Ayala A Leukocyte apoptosis and its significance in sepsis and shock J Leukoc Biol 2005 78 325 337 15817707 10.1189/jlb.0105017

[B23] Caille V Chiche JD Nciri N Berton C Gibot S Boval B Payen D Mira JP Mebazaa A Histocompatibility leukocyte antigen-D related expression is specifically altered and predicts mortality in septic shock but not in other causes of shock Shock 2004 22 521 526 15545822 10.1097/01.shk.0000143410.63698.57

[B24] Monneret G Venet F Pachot A Lepape A Monitoring immune dysfunctions in the septic patient: a new skin for the old ceremony Mol Med 2008 14 64 78 18026569 10.2119/2007-00102.Monneret PMC2078557

[B25] Nierhaus A Montag B Timmler N Frings DP Gutensohn K Jung R Schneider CG Pothmann W Brassel AK Schulte Am Esch J Reversal of immunoparalysis by recombinant human granulocyte – macrophage colony-stimulating factor in patients with severe sepsis Intensive Care Med 2003 29 646 651 12595977 10.1007/s00134-003-1666-6

[B26] Schefold JC von Haehling S Corsepius M Pohle C Kruschke P Zuckermann H Volk HD Reinke P A novel selective extracorporeal intervention in sepsis: immunoadsorption of endotoxin, interleukin 6, and complement-activating product 5a Shock 2007 28 418 425 17558345 10.1097/shk.0b013e31804f5921

[B27] Belge KU Dayyani F Horelt A Siedlar M Frankenberger M Frankenberger B Espevik T Ziegler-Heitbrock L The proinflammatory CD14^+^CD16^+^DR^++ ^monocytes are a major source of TNF J Immunol 2002 168 3536 3542 11907116 10.4049/jimmunol.168.7.3536

[B28] Ziegler-Heitbrock HW Strobel M Fingerle G Schlunck T Pforte A Blumenstein M Haas JG Small (CD14^+^/CD16^+^) monocytes and regular monocytes in human blood Pathobiology 1991 59 127 130 1715711 10.1159/000163629

[B29] Ziegler-Heitbrock HW Strobel M Kieper D Fingerle G Schlunck T Petersmann I Ellwart J Blumenstein M Haas JG Differential expression of cytokines in human blood monocyte subpopulations Blood 1992 79 503 511 1370390

[B30] Fingerle G Pforte A Passlick B Blumenstein M Strobel M Ziegler-Heitbrock HW The novel subset of CD14^+^/CD16^+ ^blood monocytes is expanded in sepsis patients Blood 1993 82 3170 3176 7693040

[B31] Shortman K Liu YJ Mouse and human dendritic cell subtypes Nat Rev Immunol 2002 2 151 161 11913066 10.1038/nri746

[B32] Onai N Obata-Onai A Schmid MA Ohteki T Jarrossay D Manz MG Identification of clonogenic common Flt3^+^M-CSFR^+ ^plasmacytoid and conventional dendritic cell progenitors in mouse bone marrow Nat Immunol 2007 8 1207 1216 17922016 10.1038/ni1518

[B33] Krug A Rothenfusser S Hornung V Jahrsdörfer B Blackwell S Ballas ZK Endres S Krieg AM Hartmann G Identification of CpG oligonucleotide sequences with high induction of IFN-alpha/beta in plasmacytoid dendritic cells Eur J Immunol 2001 31 2154 2163 11449369 10.1002/1521-4141(200107)31:7<2154::aid-immu2154>3.0.co;2-u

[B34] Efron PA Martins A Minnich D Tinsley K Ungaro R Bahjat FR Hotchkiss R Clare-Salzler M Moldawer LL Characterization of the systemic loss of dendritic cells in murine lymph nodes during polymicrobial sepsis J Immunol 2004 173 3035 3043 15322163 10.4049/jimmunol.173.5.3035

[B35] Pichyangkul S Endy TP Kalayanarooj S Nisalak A Yongvanitchit K Green S Rothman AL Ennis FA Libraty DH A blunted blood plasmacytoid dendritic cell response to an acute systemic viral infection is associated with increased disease severity J Immunol 2003 171 5571 5578 14607965 10.4049/jimmunol.171.10.5571

[B36] Scumpia PO McAuliffe PF O'Malley KA Ungaro R Uchida T Matsumoto T Remick DG Clare-Salzler MJ Moldawer LL Efron PA CD11c^+ ^dendritic cells are required for survival in murine polymicrobial sepsis J Immunol 2005 175 3282 3286 16116220 10.4049/jimmunol.175.5.3282

[B37] Guisset O Dilhuydy MS Thiebaut R Lefevre J Camou F Sarrat A Gabinski C Moreau JF Blanco P Decrease in circulating dendritic cells predicts fatal outcome in septic shock Intensive Care Med 2007 33 148 152 17091240 10.1007/s00134-006-0436-7

[B38] Hotchkiss RS Tinsley KW Swanson PE Grayson MH Osborne DF Wagner TH Cobb JP Coopersmith C Karl IE Depletion of dendritic cells, but not macrophages, in patients with sepsis J Immunol 2002 168 2493 2500 11859143 10.4049/jimmunol.168.5.2493

[B39] Levy MM Fink MP Marshall JC Abraham E Angus D Cook D Cohen J Opal SM Vincent JL Ramsay G 2001 SCCM/ESICM/ACCP/ATS/SIS International Sepsis Definitions Conference Crit Care Med 2003 31 1250 1256 12682500 10.1097/01.CCM.0000050454.01978.3B

[B40] Docke WD Hoflich C Davis KA Rottgers K Meisel C Kiefer P Weber SU Hedwig-Geissing M Kreuzfelder E Tschentscher P Nebe T Engel A Monneret G Spittler A Schmolke K Reinke P Volk HD Kunz D Monitoring temporary immunodepression by flow cytometric measurement of monocytic HLA-DR expression: a multicenter standardized study Clin Chem 2005 51 2341 2347 16214828 10.1373/clinchem.2005.052639

[B41] Wang SY Mak KL Chen LY Chou MP Ho CK Heterogeneity of human blood monocyte: two subpopulations with different sizes, phenotypes and functions Immunology 1992 77 298 303 1427982 PMC1421624

[B42] Thomas R Lipsky PE Human peripheral blood dendritic cell subsets. Isolation and characterization of precursor and mature antigen-presenting cells J Immunol 1994 153 4016 4028 7523513

[B43] Gordon S Taylor PR Monocyte and macrophage heterogeneity Nat Rev Immunol 2005 5 953 964 16322748 10.1038/nri1733

[B44] Skrzeczynska J Kobylarz K Hartwich Z Zembala M Pryjma J CD14^+^CD16^+ ^monocytes in the course of sepsis in neonates and small children: monitoring and functional studies Scand J Immunol 2002 55 629 638 12028567 10.1046/j.1365-3083.2002.01092.x

[B45] Munoz C Carlet J Fitting C Misset B Bleriot JP Cavaillon JM Dysregulation of in vitro cytokine production by monocytes during sepsis J Clin Invest 1991 88 1747 1754 1939659 10.1172/JCI115493 PMC295719

[B46] Grutz G New insights into the molecular mechanism of interleukin-10-mediated immunosuppression J Leukoc Biol 2005 77 3 15 15522916 10.1189/jlb.0904484

[B47] Tinsley KW Grayson MH Swanson PE Drewry AM Chang KC Karl IE Hotchkiss RS Sepsis induces apoptosis and profound depletion of splenic interdigitating and follicular dendritic cells J Immunol 2003 171 909 914 12847261 10.4049/jimmunol.171.2.909

[B48] Wysocka M Robertson S Riemann H Caamano J Hunter C Mackiewicz A Montaner LJ Trinchieri G Karp CL IL-12 suppression during experimental endotoxin tolerance: dendritic cell loss and macrophage hyporesponsiveness J Immunol 2001 166 7504 7513 11390504 10.4049/jimmunol.166.12.7504

[B49] Benjamim CF Lundy SK Lukacs NW Hogaboam CM Kunkel SL Reversal of long-term sepsis-induced immunosuppression by dendritic cells Blood 2005 105 3588 3595 15604223 10.1182/blood-2004-08-3251 PMC1895017

[B50] Pene F Zuber B Courtine E Rousseau C Ouaaz F Toubiana J Tazi A Mira JP Chiche JD Dendritic cells modulate lung response to *Pseudomonas aeruginosa *in a murine model of sepsis-induced immune dysfunction J Immunol 2008 181 8513 8520 19050269 10.4049/jimmunol.181.12.8513

[B51] Rieser C Papesh C Herold M Bock G Ramoner R Klocker H Bartsch G Thurnher M Differential deactivation of human dendritic cells by endotoxin desensitization: role of tumor necrosis factor-alpha and prostaglandin E_2_ Blood 1998 91 3112 3117 9558364

[B52] Abe M Tokita D Raimondi G Thomson AW Endotoxin modulates the capacity of CpG-activated liver myeloid DC to direct Th1-type responses Eur J Immunol 2006 36 2483 2493 16917958 10.1002/eji.200535767

